# Establishment and characterization of three human pluripotent stem cell lines from patients with spinocerebellar ataxia 27B (SCA27B)

**DOI:** 10.1016/j.scr.2026.104055

**Published:** 2026-06-30

**Authors:** David Pellerin, Adriana Rebelo, Mohammad Faraz Zafeer, Meghan Ditmeyer, Pablo Iruzubieta, Liedewei Van de Vondel, Yasmani Rodriguez, Elizabeth H. Jacobs, Marie-Josée Dicaire, Matt C. Danzi, Marek Napierala, Mario Saporta, Bernard Brais, Stephan Zuchner

**Affiliations:** a Dr. John T. Macdonald Foundation Department of Human Genetics and John P. Hussman Institute for Human Genomics, University of Miami Miller School of Medicine, Miami, FL, USA; b Department of Neurology and Neurosurgery, Montreal Neurological Hospital and Institute, McGill University, Montreal, QC, Canada; c Department of Neurology, University of Miami Miller School of Medicine, Miami, FL, USA; d Department of Neurology, O’Donnell Brain Institute, University of Texas Southwestern Medical Center, Dallas, TX, USA; e Department of Human Genetics, McGill University, Montreal, QC, Canada

**Keywords:** FGF14, GAA-FGF14 ataxia, Repeat expansion disorder, iPSC

## Abstract

Spinocerebellar ataxia 27B (SCA27B) is a common autosomal dominant cerebellar ataxia caused by an intronic GAA•TTC repeat expansion in the *FGF14* gene. Here, we report the generation and validation of three human induced pluripotent stem cell (iPSC) lines derived from unrelated individuals with SCA27B, including two lines carrying a monoallelic pathogenic GAA•TTC repeat expansion in *FGF14* and one line with biallelic expansions. These iPSC lines constitute a valuable resource, particularly given the predominantly neuronal expression of *FGF14*, and enable the investigation of disease mechanisms in relevant cell types following neuronal differentiation.

## Resource Table:

**Table:** 

Unique stem cell line identifier	UMi054-A UMi055-A UMi056-A
Institution	University of Miami Miller School of Medicine
Contact information of the distributor	Stephan Zuchner – SZuchner@med.miami.edu
Type of cell line	iPSC
Origin	Human
Additional human source info required for human ESC or iPSC	UMi054-A – 46, Male, French-Canadian UMi055-A – 58, Male, French-Canadian UMi056-A – 68, Male, French-Canadian
Cell Source	Dermal fibroblasts
Clonality	Clonal
Method of reprogramming	Sendai virus (CytoTune-iPS 2.0)
Genetic Feature(s)	UMi054-A – monoallelic *FGF14* (GAA•TTC)_583_ repeat expansion UMi055-A – monoallelic *FGF14* (GAA•TTC)_391_ repeat expansion UMi056-A – biallelic *FGF14* (GAA•TTC)_315_ and (GAA•TTC)_290_ repeat expansions
Evidence of the reprogramming transgene loss (including genomic copy if applicable)	RT-PCR
Associated disease	Spinocerebellar ataxia 27B (SCA27B) – OMIM #620174
Gene/locus	*FGF14* gene; chromosome 13q33.1 UMi054-A – NM_175929.3(FGF14): c.208 + 239747CTT[583] and c.208 + 239747CTT UMi055-A – NM_175929.3(FGF14): c.208 + 239747CTT[391] and c.208 + 239747CTT UMi056-A – NM_175929.3(FGF14): c.208 + 239747CTT[315] and c.208 + 239747CTT[290]
Date archived/stock date	UMi054-A: May 3rd, 2026 UMi055-A: May 4th, 2026 UMi056-A: May 4th, 2026	
Cell line repository/bank	https://hpscreg.eu/cell-line/UMi054-A https://hpscreg.eu/cell-line/UMi055-A https://hpscreg.eu/cell-line/UMi056-A
Ethical approval	Montreal Neurological Hospital Institutional Review Board: MPE-CUSM-15-915 and Institutional Review Board of the Centre Hospitalier de l’Université de Montréal: ND 02.045

## 1. Resource utility

SCA27B is an autosomal dominant ataxia caused by an intronic GAA•TTC repeat expansion in the fibroblast growth factor 14 (*FGF14*) gene (Pellerin et al., 2023). Since *FGF14* is predominantly expressed in neuronal cells (Pellerin et al., 2025), iPSCs constitute a valuable resource to enable the investigation of disease mechanisms in relevant cell types following neuronal differentiation. Here, we have established iPSC lines from three unrelated SCA27B patients.

## 2. Resource details

*FGF14* encodes an intracellular member of the fibroblast growth factor family that is widely expressed in the central nervous system, most abundantly in the cerebellum (Pellerin et al., 2025; Wang et al., 2002). It plays a key role in regulating spontaneous and evoked firing of Purkinje cells by modulating voltage-gated sodium channels at the axon initial segment (Xiao et al., 2013; Di Re et al., 2017; Lou et al., 2005). Loss of *FGF14* function in mice has been shown to cause ataxia by altering cerebellar Purkinje neuron excitability as a result of impaired sodium channels kinetics (Bosch et al., 2015; Yan et al., 2014; Shakkottai et al., 2009; Ransdell et al., 2026). Furthermore, loss-of-function variants in humans are known to underlie SCA27A (OMIM #193003), an early-onset cerebellar ataxia (Choquet et al., 2015; Dalski et al., 2005; Türkdoğan et al., 2025). In SCA27B (OMIM #620174), current evidence suggests that the intronic GAA•TTC repeat expansion in *FGF14* causes transcriptional repression, ultimately leading to Purkinje cell degeneration (Pellerin et al., 2023; Gsell et al., 2025). The restricted expression of *FGF14* complicates its study in disease-relevant models, as it requires the generation of neuronal cells.

Here, we report the generation and characterization of three iPSC lines derived from individuals with SCA27B carrying pathogenic *FGF14* GAA•TTC repeat expansions. Skin biopsies were obtained from two individuals carrying monoallelic expansions (UMi054-A and UMi055-A) and one carrying biallelic expansions (UMi056-A). The fibroblasts from these individuals had the following genotypes: 9 and 518 GAA•TTC repeats, 16 and 409 GAA⋅TTC repeats, and 295 and 304 GAA⋅TTC repeats, respectively. All patients exhibited a phenotype typical for SCA27B, characterized by late-onset, slowly progressive cerebellar ataxia preceded by episodic symptoms (Pellerin et al., 2023). Dermal fibroblasts were extracted from punch skin biopsies and reprogrammed into iPSCs using a non-integrating Sendai virus-based approach (CytoTune^™^-iPS 2.0). Multiple colonies were initially established for each individual, and clones displaying typical pluripotent stem cell morphology, characterized by a high nuclear-to-cytoplasmic ratio and well-defined colony borders, were selected and expanded under feeder-free conditions with mTeSR plus medium. The clone with the best morphology was expanded and validated as outlined below (Fig. 1A). Clearance of the Sendai viral vector was confirmed by RT-PCR at passage 10 (UMi055-A and UMi056-A) and 11 (UMi054-A) (Supplementary Fig. 1).

Expression of markers of the undifferentiated human pluripotent stem cell (hPSC) state was evaluated by quantitative PCR (qPCR) of OCT4 and SOX2 (Fig. 1B), as well as immunofluorescence staining for OCT4, SOX2, TRA-1–60, and SSEA4 (Fig. 1C). Furthermore, all lines were confirmed to be free of mycoplasma contamination (Supplementary Fig. 2) and exhibited normal karyotypes (46,XY for all lines), supporting their genomic stability (Fig. 1E). Short tandem repeat (STR) analysis confirmed that iPSCs were not contaminated and matched the donor fibroblasts. Repeat sizing of the *FGF14* locus confirmed the presence of expanded GAA•TTC alleles in all lines, with repeat lengths of 9 and 583 (UMi054-A), 16 and 391 (UMi055-A), and 290 and 315 (UMi056-A) (Supplementary Fig. 3). As a result of *FGF14* repeat instability, we observed slight differences in repeat expansion lengths between matched fibroblasts and iPSC clones, as reported previously (Pellerin et al., 2025).

To assess differentiation capacity, *in vitro* directed trilineage differentiation was performed at passage 14 (UMi054-A) and 15 (UMi055-A and UMi056-A) (Fig. 1D). Immunofluorescence staining demonstrated expression of lineage-specific markers, including PAX6 and nestin for ectoderm, brachyury and smooth muscle actin (SMA) for mesoderm, and SOX17 and FOXA2 for endoderm, confirming the ability of all iPSC lines to give rise to derivatives of the three germ layers. Imaging of both undifferentiated hPSC state and trilineage differentiation markers was conducted at 40 × magnification using a Zeiss LSM 980 confocal microscope. Table 1 summarizes the characterization and validation of all three lines (see also Table 2).

## 3. Materials and methods

### 3.1. iPSC reprogramming and culture

Dermal fibroblasts were obtained from skin punch biopsies of three individuals with SCA27B. Fibroblasts were maintained in DMEM supplemented with GlutaMAX (Gibco, cat. 10569010), 10% (v/v) fetal bovine serum (Gibco, cat. 26140079), 100 U/ml penicillin–streptomycin (Gibco, cat. 15140122), and 2.5 μg/ml amphotericin B (Gibco, cat. 15290018). Transduction was carried out using the CytoTune^™^-iPS 2.0 Sendai Reprogramming Kit (ThermoFisher, cat. A16517) according to the manufacturer's instructions. Emerging iPSC colonies were manually picked and transferred onto Vitronectin-coated plates (0.5 μg/cm^2^; Gibco, cat. A400457) in Essential 8^™^ Medium (ThermoFisher, cat. A1517001). The following day, a half-medium change to mTeSR^™^ Plus (STEMCELL Technologies, cat. 100–0276) was performed, with subsequent feeds consisting of full mTeSR^™^ Plus changes. At 70–80% confluence, iPSCs were passaged as clumps using 0.5 mM EDTA in D-PBS (1:4 to 1:6 split ratio) with 10 μM ROCK inhibitor Y-27632 (STEMCELL Technologies, cat. 72304) added for 24 h. The medium was changed daily. All cells were cultured at 37°C in a humidified atmosphere of 5% CO_2_.

### 3.2. Sendai vector clearance

Clearance of residual reprogramming vector in iPSC lines was assessed by RT-PCR at passage 10 (UMi055-A and UMi056-A) and 11 (UMi054-A). Cells were homogenized using QIAshredder spin columns (Qiagen, cat. 79656), and total RNA was extracted with the RNeasy Plus Mini Kit (Qiagen, cat. 74136). Complementary DNA (cDNA) was synthesized using the QuantiTect Reverse Transcription Kit (Qiagen, cat. 205313) according to the manufacturer’s protocol (genomic DNA elimination reaction at 42°C for 2 min, followed by reverse transcription at 42°C for 15 min, then by inactivation at 95°C for 3 min). PCR amplification of cDNA was performed using Platinum^™^ II Taq Hot-Start DNA Polymerase (Invitrogen, cat. 14000013). PCR amplification was performed using a touchdown cycling protocol: an initial denaturation at 94°C for 3 min (2 cycles), followed by a touchdown phase of 12 cycles (2 cycles per annealing temperature) with denaturation at 94°C for 10 s, annealing at decreasing temperatures from 65°C to 55°C (10 s each), and extension at 72°C for 40 s, then 32 additional cycles at 94°C for 10 s, 55°C for 30 s, and 72°C for 40 s, with a final hold at 4°C. Amplicons were resolved on a 1% agarose gel containing 0.5 μg/ml ethidium bromide (Invitrogen, cat. 15585011).

### 3.3. Karyotyping and STR analysis

G-band karyotype analysis was performed at passage 9 (UMi055-A and UMi056-A) and 12 (UMi054-A) by WiCell Research Institute (Madison, WI, USA), with 20 metaphase spreads examined per line. Short tandem repeat (STR) profiling was performed at passage 9 (UMi055-A and UMi056-A) and 12 (UMi054-A) by WiCell Research Institute using the PowerPlex^®^ 16 HS System (Promega, cat. DC2101). Identity of each iPSC line was confirmed by 100% allelic concordance with its parental fibroblast across all 15 loci and amelogenin.

### 3.4. Mycoplasma testing

Mycoplasma contamination was assessed at passage 9 (UMi055-A and UMi056-A) and 12 (UMi054-A) by WiCell Research Institute using the EZ-PCR^™^ Mycoplasma Detection Kit (Sartorius, cat. 20–700–20), which included a positive and negative control.

### 3.5. FGF14 GAA•TTC repeat genotyping

Genomic DNA was extracted using the DNeasy Blood & Tissue Kit (Qiagen, cat. 69506). *FGF14* intronic GAA•TTC repeat length was determined by long-range and bidirectional repeat-primed PCRs following the protocol described by Bonnet et al. (2023).

### 3.6. Analysis of markers of the undifferentiated hPSC state

iPSC colonies were assessed for expression of markers of the undifferentiated hPSC state by qPCR and immunofluorescence at passage 12 (UMi055-A and UMi056-A) and 14 (UMi054-A). cDNA was synthesized as described above and qPCR was performed using the PowerUp SYBR Green master mix (ThermoFisher, cat. A25742) on an Applied Biosystems QuantStudio 12 K Flex Real-Time PCR System using the fast cycling protocol (95°C for 20 s, followed by 40 cycles of 95°C for 1 s and 60°C for 20 s) in a 10 μl reaction volume. All qPCR experiments were performed in triplicate, and data are presented as mean ± standard deviation. Gene expression levels in the iPSC lines were compared to those in a validated control iPSC line (Ku et al., 2010). Expression was normalized to the geometric mean of two housekeeping genes (*ACTB* and *GAPDH*). In preparation for immunofluorescence, cells were fixed with 4% paraformaldehyde (Invitrogen, cat. J19943.K2) for 15 min, permeabilized with 0.1% Triton X-100 (ThermoFisher, cat. HFH10) for 15 min, and blocked with 2% bovine serum albumin (Sigma-Aldrich, cat. A9418) for 1 h at room temperature. Primary antibodies were applied overnight at 4°C. Following three PBS washes, cells were incubated with the appropriate fluorophore-conjugated secondary antibodies for 1 h at room temperature, washed three times, and counterstained with 4′,6-diamidino-2-phenylindole (DAPI; Invitrogen, cat. 62248) for 15 min. Images were acquired using a Zeiss LSM 980 confocal microscope.

### 3.7. Trilineage differentiation

Directed trilineage differentiation was performed on iPSC lines at passage 14 (UMi054-A) and 15 (UMi055-A and UMi056-A) using the STEMdiff^™^ Trilineage Differentiation Kit (STEMCELL Technologies, cat. 05230) according to the manufacturer's protocol. iPSCs were dissociated into a single-cell suspension using Gentle Cell Dissociation Reagent (STEMCELL Technologies, cat. 100–0485) and plated onto Matrigel-coated plates (Corning, cat. 354277) at the recommended density. At the end of each lineage-specific differentiation protocol, cells were processed for immunofluorescence as described above.

## Supplementary Material

1

## Figures and Tables

**Fig. 1.**
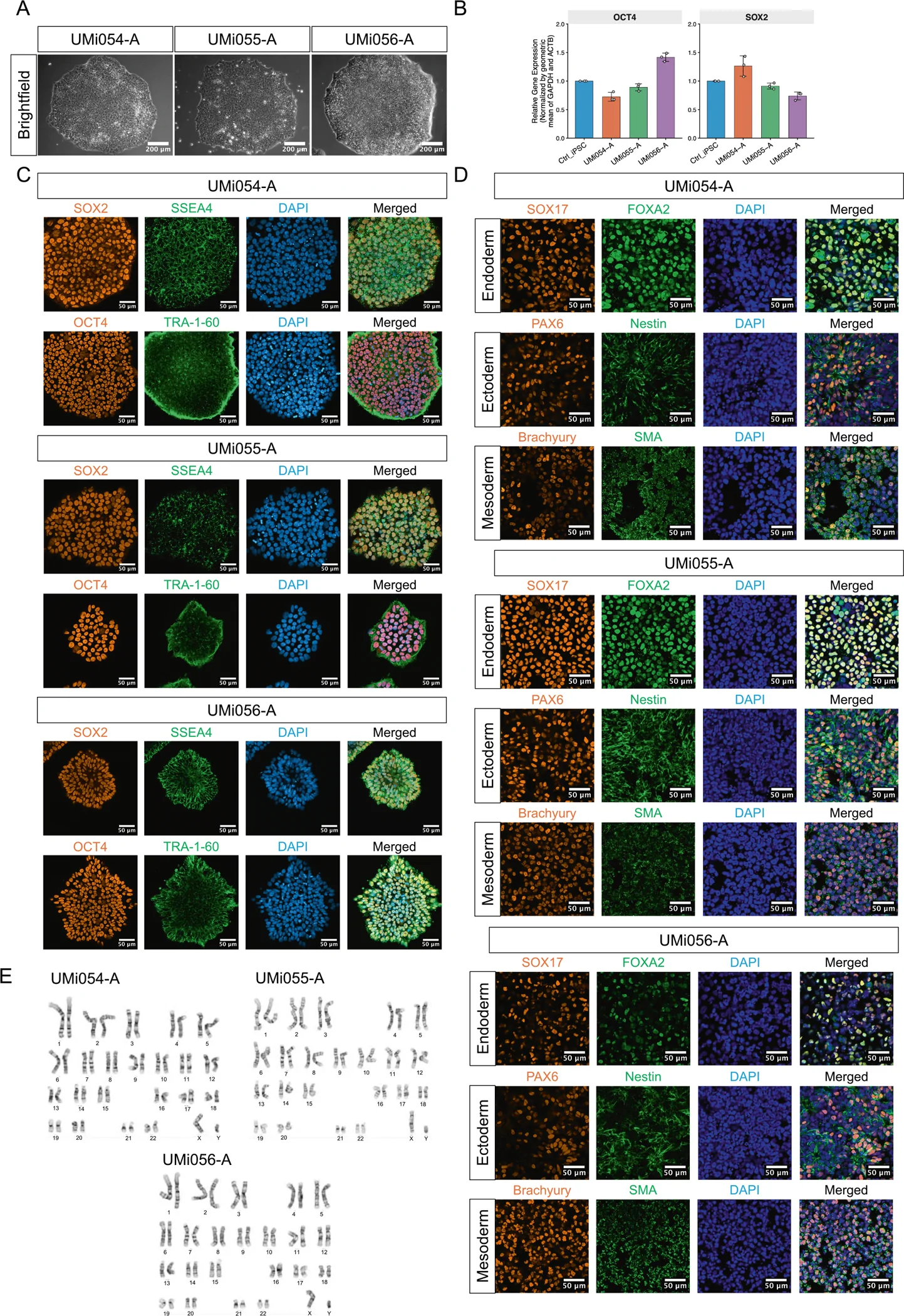


**Table 1:** Characterization and validation.

Classification	Test	Result	Data

**Morphology**	Photography Bright field	Normal	Fig. 1 panel A
**Characterization of undifferentiated state**	Marker expression analysis RT-qPCR Immunofluorescence	Positive gene expression for markers of the undifferentiated hPSC state: SOX2 and OCT4 Positive staining for markers of the undifferentiated hPSC state: OCT4, SOX2, SSEA4, and TRA-1–60	Fig. 1 panel B Fig. 1 panel C
**Genomic stability**	Karyotyping by G-banding	UMi054-A: 46, XY UMi055-A: 46, XY UMi056-A: 46, XY	Fig. 1 panel E
**Cell line identity verification**	Microsatellite PCR (mPCR) OR STR analysis	N/A	N/A
		iPSCs match derivative fibroblasts at 15 loci + amelogenin	Submitted in archive with the journal
**Mutation analysis**	Long-range PCR and repeat-primed PCR	UMi054-A: monoallelic *FGF14* (GAA•TTC)_583_ expansion	Supplementary Fig. 3
		UMi055-A: monoallelic *FGF14* (GAA•TTC)_391_ expansion	
		UMi056-A: biallelic *FGF14* (GAA•TTC)_290_ and (GAA•TTC)_315_ expansions	
**Microbiology and virology**	Mycoplasma	Mycoplasma testing by RT-PCR: Negative for all three lines	Supplementary Fig. 2
**Characterization of the differentiated state**	Directed tri-lineage differentiation. Expression of these markers has to be demonstrated at mRNA (RT PCR) or protein (IF) levels, at least 2 markers need to be shown per germ layer	Positive via immunofluorescence Ectoderm: PAX6, Nestin Endoderm: SOX17, FOXA2 Mesoderm: Brachyury, SMA	Fig. 1 panel D
**Donor screening (OPTIONAL)**	HIV 1 + 2 Hepatitis B, Hepatitis C	N/A	N/A
**Genotype additional info (OPTIONAL)**	Blood group genotyping	N/A	N/A
	HLA tissue typing	N/A	N/A

**Table 2:** Reagent details.

Antibodies used for immunocytochemistry				
	Antibody	Dilution	Company Cat#	RRID

Markers of undifferentiated hPSC state	Mouse anti-TRA-1–60(S)	1:2000	Cell Signaling Technology Cat#4746	AB_2119059
	Rabbit anti-SOX2	1:100	Millipore Cat#AB5603	AB_2286686
	Mouse anti-SSEA4	1:500	Invitrogen Cat#53–8843-42	AB_10609335
	Rabbit Anti-OCT4	1:200	Cell Signaling Technology Cat#2750	AB_823583
Differentiation Markers – Ectoderm	Rabbit anti-PAX6 (D3A9V)	1:200	Cell Signaling Technology Cat#60433	AB_2797599
	Mouse anti-Nestin, Clone 10C2	1:1000	STEMCELL Technologies Cat#60091	AB_2650581
Differentiation Markers – Mesoderm	Rabbit anti-Brachyury (D2Z3J)	1:1000	Cell Signaling Technology Cat#81694	AB_2799983
	Mouse anti-SMA (B4)	1:100	Santa Cruz Biotechnology Cat#sc-53142	AB_2273670
Differentiation Markers – Endoderm	Rabbit anti-SOX17 (D1T8M)	1:3200	Cell Signaling Technology Cat#81778	AB_2650582
	Mouse anti-FOXA2	1:100	Abcam Cat#AB60721	AB_941632
Secondary antibodies	Alexa Fluor^™^ 555 goat anti-rabbit IgG (H + L)	1:500	Invitrogen Cat#A21428	AB_2535849
	Alexa Fluor^™^ 488 goat anti-mouse IgG (H + L)	1:500	Invitrogen Cat#A11001	AB_2534069
Cell nuclei	DAPI	1:1000	Invitrogen Cat#62248	AB_2307445
Primers	Target	Size of amplification product	Forward/Reverse primer (5′-3′)	

Genomic integration analysis (RT-PCR)	Sendai viral vector (SeV)	181 bp	Fw: GGATCACTAGGTGATATCGAGC Rv: ACCAGACAAGAGTTTAAGAGATATGTATC	
	Beta-actin	409 bp	Fw: CATGGAGAAAATCTGGCACCAC Rv: GCACAGCTTCTCCTTAATGTCAC	
Markers of undifferentiated hPSC state (qPCR)	OCT4 SOX2	142 bp 126 bp	Fw: AGCGAACCAGTATCGAGAAC Rv: TTACAGAACCACACTCGGAC	
			Fw: AGCTACAGCATGATGCAGGA Rv: GGTCATGGAGTTGTACTGCA	
Housekeeping genes (qPCR)	Beta-actin GAPDH	91 bp 66 bp	Fw: ATTGGCAATGAGCGGTTC Rv: TGAAGGTAGTTTCGTGGATGC Fw: AGCCACATCGCTCAGACAC Rv: GCCCAATACGACCAAATCC	
Long-range PCR	FGF14		Fw: TGCAAATGAAGGAAAACTCTT Rv: CAATGATGAATTAAGCAGTTCC	
Fluorescent long-range PCR	FGF14		Fw: 6-FAM-TGCAAATGAAGGAAAACTCTT Rv: CAATGATGAATTAAGCAGTTCC	
5′ Repeat-primed PCR	FGF14		Fw: TGCCCACATAGAGCTTAGTCT Rv: CACGACGTTGTAAAACGAC- GAAGAAGAAGAAGAAGAAGAA M13-FAM: 6-FAM- CACGACGTTGTAAAACGAC	
3′ Repeat-primed PCR	FGF14		F: TGCAAATGAAGGAAAACTCTT R: CACGACGTTGTAAAACGAC- TTCTTCTTCTTCTTCTTCTTC M13-FAM: 6-FAM- CACGACGTTGTAAAACGAC	

## Data Availability

Data will be made available on request.

## Appendix A. Supplementary data

Supplementary data to this article can be found online at https://doi.org/10.1016/j.scr.2026.104055.
