# Supplementary material for: Establishment and characterization of three human pluripotent stem cell lines from patients with spinocerebellar ataxia 27B (SCA27B)
Source: Stem Cell Res. Author manuscript; Available in PMC 2026 Oct 17. (PMC13675862; doi:10.1016/j.scr.2026.104055)
Supplement: Supplementary file 1 [file NIHMS2209964-supplement-1.pdf]

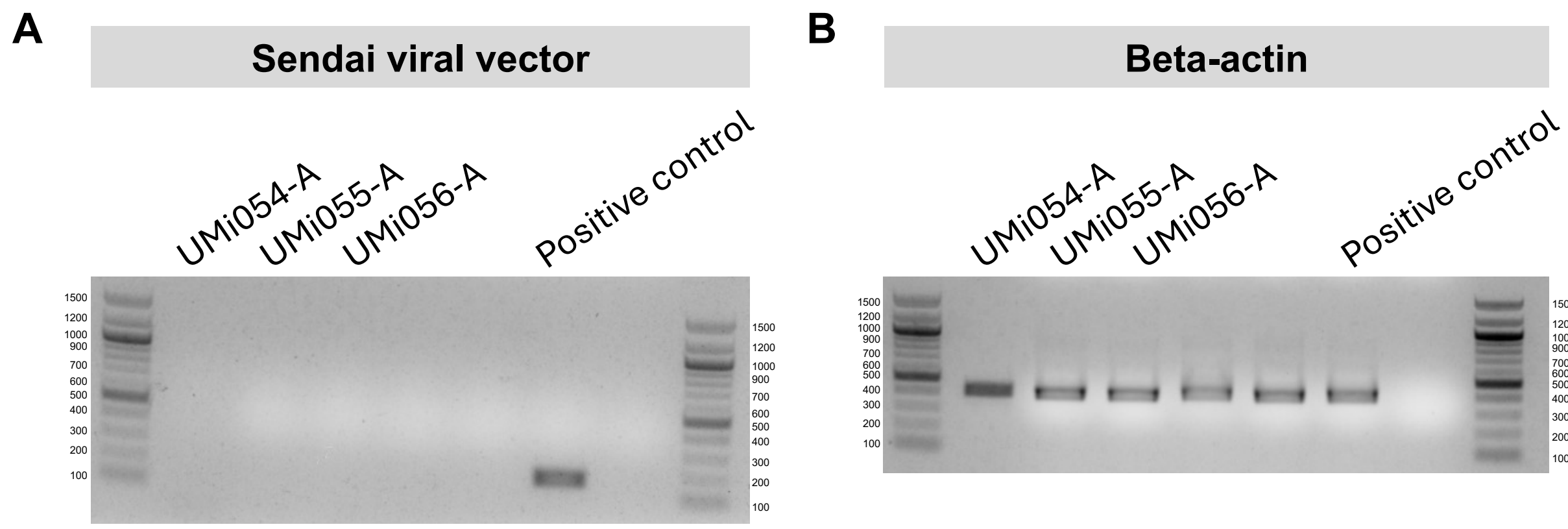

Supplementary Figure 1. Absence of Sendai viral reprogramming vector in iPSC lines.

RT-PCR-based detection of (A) Sendai viral vector and (B) beta-actin (control) in UMi054-A, UMi055-A, and UMi056-A iPSC lines. No amplification band is detected for the Sendai viral vector in any of the three iPSC lines, confirming complete clearance of the reprogramming vector. Beta-actin amplification confirms cDNA quality across all samples. A Sendai virus-infected cell line was included as a positive control.

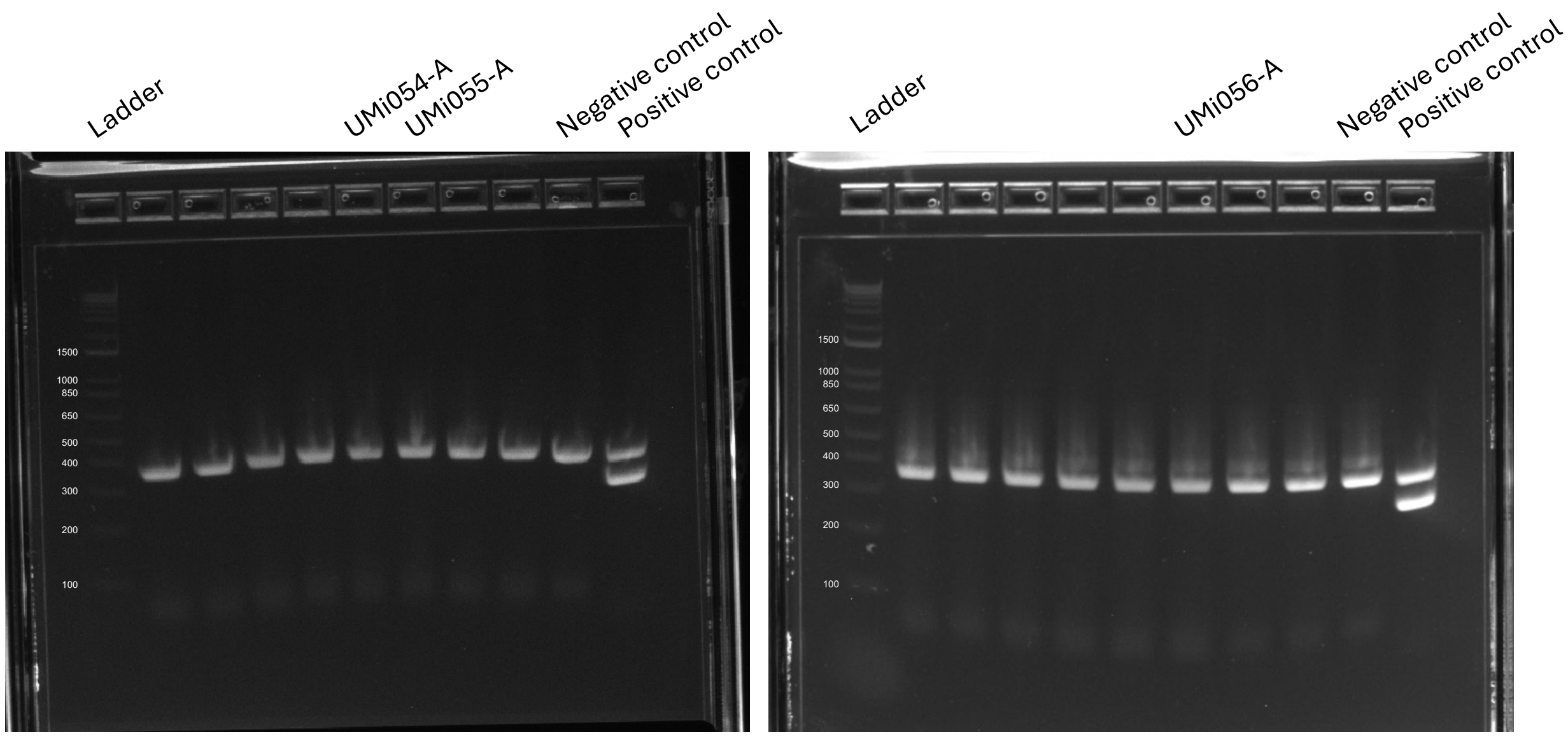

Supplementary Figure 2. Mycoplasma testing of iPSC lines.

PCR-based mycoplasma detection performed on UMi054-A and UMi055-A (left) and UMi056-A (right) iPSC lines using the EZ-PCR™ Mycoplasma Detection Kit (Sartorius), resolved on a 2% agarose E-gel with SYBR Safe staining. All samples are negative for mycoplasma contamination, as evidenced by the absence of the 270 bp band. Negative and positive controls were included.

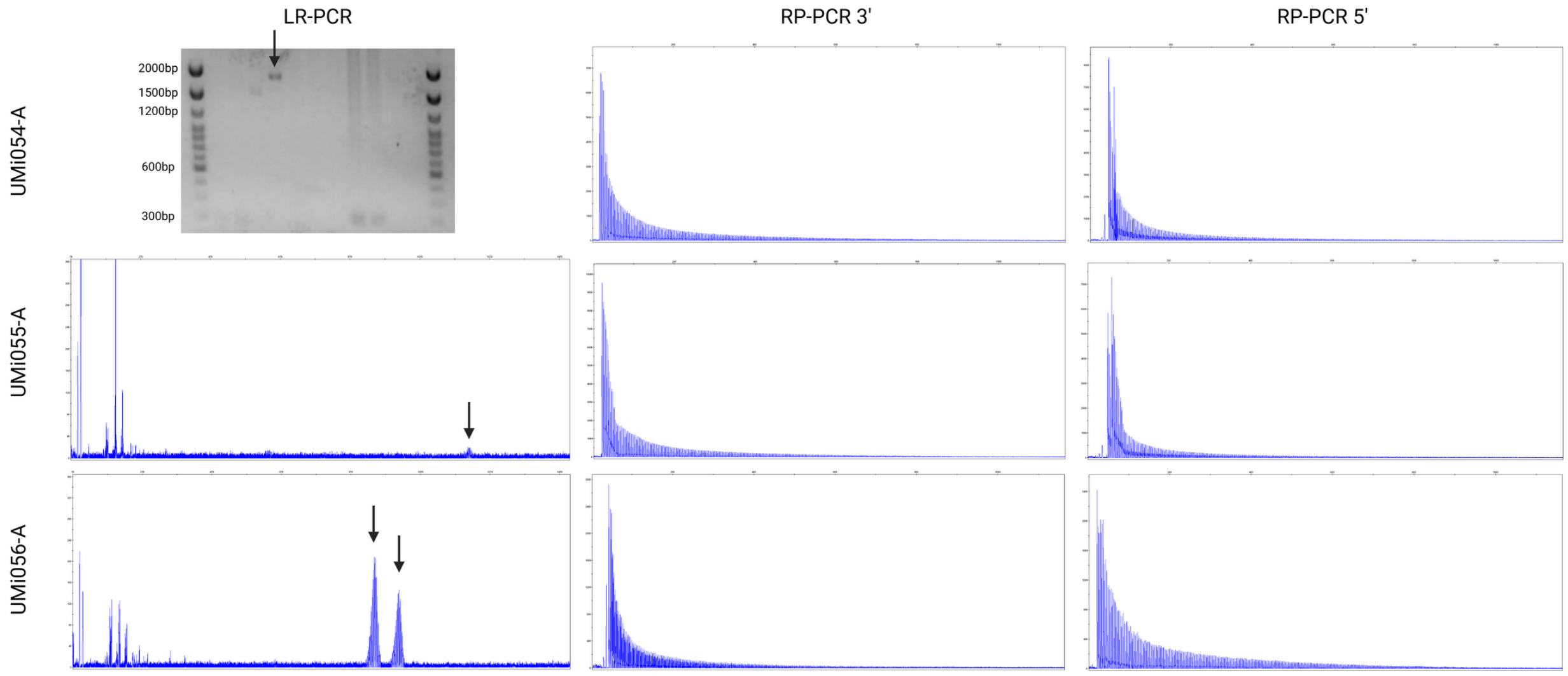

Supplementary figure 3. *FGF14* repeat genotyping of SCA27B iPSC lines.

Long-range PCR (LR-PCR) and repeat-primed PCR (RP-PCR) were performed to characterize the *FGF14* intronic GAA•TTC repeat in each iPSC line. RP-PCR was performed with primers anchored at either the 3′ or 5′ flanking region of the repeat (RP-PCR 3′ and RP-PCR 5′, respectively).

WiCell

Chromosome Analysis Report: 106884

Date Reported: ***April 20, 2025***
Cell Line: ***UMi054-A***
Submitted Passage #: ***None Given***
Date of Sample: ***4/9/2025***
Specimen: ***Human IPSC***
Results: ***46,XY***

Cell Line Sex: ***Male***
Reason for Testing: ***None Given***
Investigator: ***Adriana Rebelo, University of Miami***

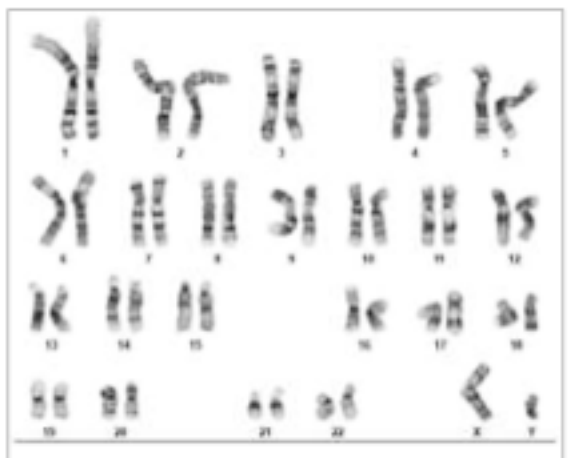

Cell: ***23***
Slide: ***G01***
Slide Type: ***Karyotype***

Total Counted: ***20***
Total Analyzed: ***8***
Total Karyogrammed: ***4***
Band Resolution: ***425 - 475***

Interpretation:
This is a normal karyotype; no clonal abnormalities were detected at the stated band level of resolution.

Completed by: ***Dawn Davis, CG(ASCP)***
Reviewed and Interpreted by: ***Justin Schleede, PhD, FACMG***

For internal use only

Date:________________________ Sent By:____ Sent To:_____________________ QC Review By: ____

Limitations: This assay allows for microscopic visualization of numerical and structural chromosome abnormalities. The size of structural abnormality that can be detected is >3-10Mb, dependent upon the G-band resolution obtained from this specimen. For the purposes of this report, band level is defined as the number of G-bands per haploid genome. It is documented here as "band level", i.e., the range of bands determined from the four karyograms in this assay. Detection of heterogeneity of clonal cell populations in this specimen (i.e.,mosaicism) is limited by the number of metaphase cells examined, documented here as "# of cells counted".

This assay was conducted solely for listed investigator/institution. The results of this assay are for research use only. Unless otherwise mutually agreed in writing, the services provided to you hereunder by WiCell Research Institute, Inc. ("WiCell") are governed solely by WiCell's Terms and Conditions of Service, found at www.wicell.org/privacyandterms. Any terms you may attach to a purchase order or other document that are inconsistent, add to, or conflict with WiCell's Terms and Conditions of Service are null and void and of no legal force or effect.

Supplementary figure 4. ***Chromosome analysis report of iPSC line UMi054-A*.**

WiCell

Chromosome Analysis Report: 106885

Date Reported: ***April 20, 2025***
Cell Line: ***UMi055-A***
Submitted Passage #: ***None Given***
Date of Sample: ***4/9/2025***
Specimen: ***Human IPSC***
Results: ***46,XY***

Cell Line Sex: ***Male***
Reason for Testing: ***None Given***

Investigator: ***Adriana Rebelo, University of Miami***

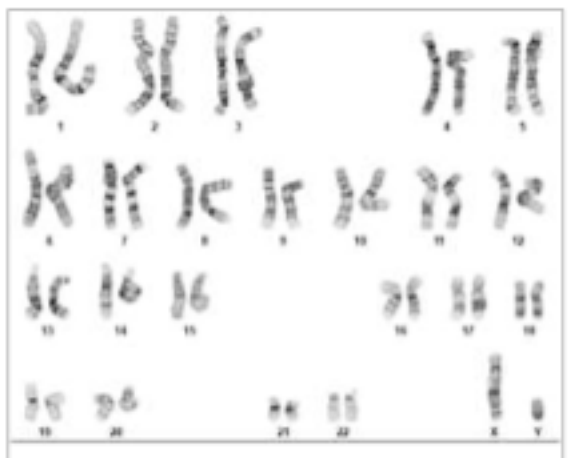

Cell: ***30***
Slide: ***G03***
Slide Type: ***Karyotype***

Total Counted: ***20***
Total Analyzed: ***8***
Total Karyogrammed: ***4***
Band Resolution: ***450 - 525***

Interpretation:
This is a normal karyotype; no clonal abnormalities were detected at the stated band level of resolution.

Completed by: ***Timm Gonzales, CG(ASCP)***
Reviewed and Interpreted by: ***Justin Schleede, PhD, FACMG***

For internal use only

Date:______________________ Sent By:____ Sent To:____________________ QC Review By: ____

Limitations: This assay allows for microscopic visualization of numerical and structural chromosome abnormalities. The size of structural abnormality that can be detected is >3-10Mb, dependent upon the G-band resolution obtained from this specimen. For the purposes of this report, band level is defined as the number of G-bands per haploid genome. It is documented here as "band level", i.e., the range of bands determined from the four karyograms in this assay. Detection of heterogeneity of clonal cell populations in this specimen (i.e.,mosaicism) is limited by the number of metaphase cells examined, documented here as "# of cells counted".

This assay was conducted solely for listed investigator/institution. The results of this assay are for research use only. Unless otherwise mutually agreed in writing, the services provided to you hereunder by WiCell Research Institute, Inc. ("WiCell") are governed solely by WiCell's Terms and Conditions of Service, found at www.wicell.org/privacyandterms. Any terms you may attach to a purchase order or other document that are inconsistent, add to, or conflict with WiCell's Terms and Conditions of Service are null and void and of no legal force or effect.

Supplementary figure 5. ***Chromosome analysis report of iPSC line UMi055-A*.**

WiCell

Chromosome Analysis Report: 105989

Date Reported: February 25, 2025
Cell Line: UMi056-A
Submitted Passage #: None Given
Date of Sample: 2/18/2025
Specimen: Human IPSC
Results: 46,XY

Cell Line Sex: Male
Reason for Testing: None Given
Investigator: Adriana Rebelo, University of Miami

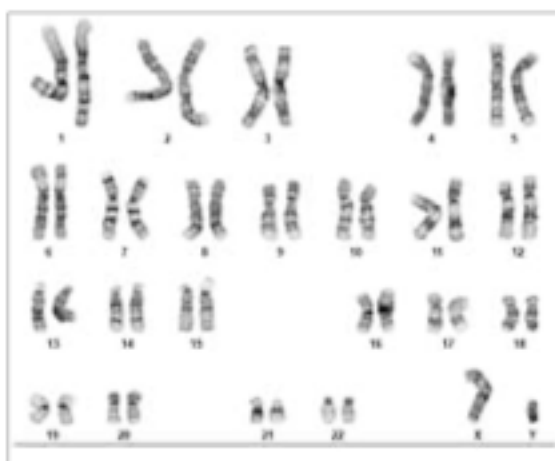

Cell: 68
Slide: G03
Slide Type: Karyotype

Total Counted: 20
Total Analyzed: 8
Total Karyogrammed: 4
Band Resolution: 425 - 475

Interpretation:
This is a normal karyotype; no clonal abnormalities were detected at the stated band level of resolution.

Completed by: Jennifer Pecos, CG(ASCP)
Reviewed and Interpreted by: Justin Schleede, PhD, FACMG

For internal use only

Date:_______________________ Sent By:____ Sent To:_____________________ QC Review By: ____

Limitations: This assay allows for microscopic visualization of numerical and structural chromosome abnormalities. The size of structural abnormality that can be detected is >3-10Mb, dependent upon the G-band resolution obtained from this specimen. For the purposes of this report, band level is defined as the number of G-bands per haploid genome. It is documented here as "band level", i.e., the range of bands determined from the four karyograms in this assay. Detection of heterogeneity of clonal cell populations in this specimen (i.e.,mosaicism) is limited by the number of metaphase cells examined, documented here as "# of cells counted".

This assay was conducted solely for listed investigator/institution. The results of this assay are for research use only. Unless otherwise mutually agreed in writing, the services provided to you hereunder by WiCell Research Institute, Inc. ("WiCell") are governed solely by WiCell's Terms and Conditions of Service, found at www.wicell.org/privacyandterms. Any terms you may attach to a purchase order or other document that are inconsistent, add to, or conflict with WiCell's Terms and Conditions of Service are null and void and of no legal force or effect.

Supplementary figure 5. ***Chromosome analysis report of iPSC line UMi056-A*.**
